# A virtual physicist framework integrating dose prediction and large language models for automated treatment planning

**DOI:** 10.1002/acm2.70904

**Published:** 2026-11-05

**Authors:** Heling Zhu, Junqiang Li, Yongguang Liang, Wenbo Li, Yiming Zhang, Wei Zhang, Bo Yang, Qichao Zhou

**Affiliations:** ^1^ Department of Radiation Oncology Peking Union Medical College Hospital, Chinese Academy of Medical Sciences and Peking Union Medical College Beijing China; ^2^ Manteia Technologies Co., Ltd Xiamen Fujian China; ^3^ UMass Chan Medical School Worcester Massachusetts USA

**Keywords:** automated planning, dose prediction, large language model, virtual physicist

## Abstract

**Background:**

Automated radiotherapy treatment planning has benefited from data‐driven approaches; however, achieving consistently high plan quality across patients remains challenging. Dose prediction models provide patient‐specific priors but lack adaptive optimization, whereas large language models (LLMs) offer strong reasoning capabilities but raise concerns regarding stability and safety in clinical use.

**Purpose:**

To develop and validate a fully automated VMAT treatment planning framework integrating dose prediction and LLM‐driven dynamic optimization, termed the Virtual physicist (VP), and to evaluate its dosimetric performance, plan complexity, and consistency relative to manual and other automated planning strategies.

**Methods:**

A total of 70 cervical cancer patients previously treated with VMAT were retrospectively collected. The dataset was divided into 50 cases for training, 10 for validation, and 10 for testing. The input data consisted of patients’ planning CT images with contoured planning target volumes (PTVs) and organs at risk (OARs). The outputs included predicted three‐dimensional dose distributions (upper‐, middle‐, and lower‐dose bands) and automatically generated VMAT treatment plans with corresponding dosimetric and plan complexity metrics. The VP framework was implemented in the MOZI treatment planning system via a Python scripting interface. A dose‐band prediction model was used to initialize patient‐specific optimization constraints. During optimization, an LLM iteratively adjusted objectives and weights based on real‐time dosimetric feedback, subject to a rule‐based safety engine enforcing clinical and physical constraints. Plans generated using dose prediction only (DP‐only), LLM only (LLM‐only), and combined dose prediction with LLM (DP + LLM) were compared with clinically delivered manual plans.

**Results:**

The DP + LLM strategy significantly improved sparing of the bladder and bilateral femoral heads compared with all three other strategies and significantly reduced rectal doses relative to the DP‐only and manual plans (*P* < 0.05), while maintaining PTV Dmean, HI, CI, and GI comparable to those of the manual plans (*P* > 0.05). For dual‐layer MLC plan complexity, DP + LLM produced a more balanced modulation pattern than manual planning, with lower distal‐layer complexity—significant for d‐C/A and d‐MCS but not d‐BA—and higher proximal‐layer complexity across all three metrics. Compared with DP‐only and LLM‐only plans, DP + LLM showed a consistent trend toward greater complexity in most metrics, with significant differences in several metrics. In addition, the automated strategies generally showed smaller standard deviations in complexity metrics, indicating improved planning consistency.

**Conclusion:**

Integrating dose prediction with LLM‐driven dynamic optimization enables a robust and fully automated planning workflow, improving OAR sparing and planning consistency without compromising target dose quality, and demonstrates strong potential for clinically applicable intelligent radiotherapy planning.

## 1 INTRODUCTION

The design and optimization of treatment plans are critical steps in the radiotherapy workflow. A high‐quality plan must ensure adequate dose coverage of the planning target volume (PTV) while minimizing radiation exposure to surrounding organs at risk (OARs). Plan quality and consistency directly influence clinical effectiveness and normal tissue sparing.^1^Conventionally, treatment planning relies on radiation dosimetrists or medical physicists who iteratively adjust optimization parameters based on individual experience and trial‐and‐error procedures. This process is inherently complex, time‐consuming, and susceptible to inter‐planner variability, which can lead to inconsistencies in plan quality.^2^With the widespread clinical adoption of advanced techniques such as volumetric modulated arc therapy (VMAT), treatment planning has progressively transitioned from experience‐driven practice toward data‐driven strategies. Nevertheless, achieving the generation of high‐quality, reproducible, and highly automated radiotherapy plans remains a central challenge in medical physics research.

To address these challenges, a variety of automated radiotherapy treatment planning approaches has been developed in recent years, among which multi‐criteria optimization (MCO) and knowledge‐based planning (KBP) are representative methods. MCO simultaneously optimizes multiple competing objective functions and generates a set of alternative plans within the Pareto‐optimal solution space, allowing medical physicists and clinicians to interactively explore trade‐offs among dosimetric objectives according to clinical preferences.^3^, ^4^, ^5^In contrast, KBP learns from large cohorts of high‐quality historical plans to establish dose–geometry relationship models using statistical or machine learning techniques. These models enable the prediction of patient‐specific dose objectives and optimization parameters for new cases, thereby reducing planning time and manual effort, decreasing dependence on individual experience, and improving overall plan consistency.^6^, ^7^, ^8^

With the continuous advancement of artificial intelligence (AI) technology, deep learning‐based dose prediction models integrated with the KBP framework have provided more efficient and accurate solutions for automated radiotherapy planning. These dose prediction models, which utilize patients’ computed tomography (CT) images, can provide optimal dose distributions achievable for the PTV and OARs prior to the initiation of optimization. This reduces the need for manual trial‐and‐error steps during optimization, lowering workload while improving plan consistency to a certain extent.^9^, ^10^, ^11^, ^12^, ^13^Although deep learning‐based models demonstrate relatively high predictive accuracy, the heterogeneity of patient data and the need for dynamic decision‐making must be considered during actual plan optimization. Relying solely on predicted doses to set optimization constraints often fails to yield optimal results. Furthermore, manual adjustments based on real‐time conditions during the iterative optimization process significantly undermine the automation of the workflow.

Meanwhile, in recent years, large language models (LLMs) have exhibited strong reasoning and interaction capabilities in target volume delineation, image processing, and clinical decision support for radiotherapy.^14^, ^15^, ^16^, ^17^The latest direction in radiotherapy automation has begun exploring the use of LLMs as “intelligent optimization assistants,” which dynamically adjust objective functions and optimization parameters based on real‐time dose feedback during the optimization process. For instance, Wang et al. (2025) proposed GPT‐Plan, a GPT‐4–based multi‐agent system that simulates collaborative workflows between dosimetrists and physicists, integrates historical plan retrieval, and implements a two‐stage self‐reflection mechanism to achieve automated iterative radiotherapy plan optimization, demonstrating the feasibility of LLM‐driven planning.^18^Wei et al. (2025) evaluated three LLMs for automated radiotherapy planning in cervical cancer and found that two generated clinically acceptable VMAT plans while one failed due to hallucinations, providing preliminary evidence for the potential of LLMs to enhance planning efficiency.^19^However, relying solely on LLMs for radiotherapy plan optimization still has limitations: LLMs lack support from personalized dose prior information, and their parameter adjustments mostly rely on general templates or statistical patterns of historical plans. This may prolong the iterative optimization time or lead the optimization direction away from the optimal path. Additionally, the hallucination phenomenon observed in some LLMs may result in unreasonable parameter settings, further compromising the dosimetric quality and clinical reliability of the plans. These shortcomings limit the optimization efficiency and stability of LLMs when used independently.

Recently, Maniscalco et al. established a multimodal TPS‐integrated compound AI platform that combined multi‐agent LLM orchestration, directive‐conditioned three‐dimensional dose prediction, and retrieval‐augmented institutional knowledge retrieval, demonstrating fully autonomous treatment planning across brain, lung, and prostate cases.^20^Building on these advances, the present study shifts the focus from establishing end‐to‐end platform feasibility to a controlled, component‐level evaluation of how dose prediction and LLM‐driven optimization contribute to automated treatment planning. We present a fully automated treatment planning framework that integrates dose‐band prediction with LLM‐driven dynamic optimization. The primary contribution of this work is its controlled three‐way comparison of DP‐only, LLM‐only, and combined DP + LLM strategies within the same TPS and patient cohort, which enables the individual and synergistic contributions of patient‐specific dose priors and LLM‐based adaptive optimization to be disentangled. In addition, the proposed VP framework incorporates a rule‐based safety engine and is evaluated using both detailed dosimetric endpoints and dual‐layer MLC complexity metrics in cervical cancer VMAT. These analyses provide component‐level evidence for the design and clinical translation of intelligent automated planning systems.

## 2 METHODS AND MATERIALS

### 2.1 Data collection

A retrospective cohort of 70 cervical cancer patients previously treated with VMAT on a Halcyon linear accelerator was randomly selected for this study. Only patients whose upper PTV boundary was located below the kidneys were included, and those with lymph node metastases were excluded. These criteria were applied to reduce anatomical and dosimetric heterogeneity, as extended‐field or node‐positive cases involve distinct optimization objectives and dose patterns that require additional training data for robust dose prediction. All patients were positioned supine and immobilized using thermoplastic body masks during simulation. CT scans were acquired using GE or Philips Big Bore scanners with a slice thickness of 5 mm.

All treatments employed 6 MV flattening filter–free (FFF) X‐rays with a maximum dose rate of 800 MU/min. Prescription doses ranged from 36.0 Gy–50.4 Gy with a uniform fractionation of 1.8 Gy, and all plans were required to cover the 95% prescription of the PTV. OARs considered included the bladder, small intestine, rectum, spinal cord, and left and right femoral heads. For each patient, the dataset comprised CT images with contoured PTV and OARs. The manually generated clinical plans were created in the Eclipse treatment planning system (TPS), whereas all automated plans were generated in the MOZI TPS (Manteia Medical Technologies, China). For both systems, the dose calculation grid size was 0.25 cm × 0.25 cm × 0.25 cm. The manual plans in Eclipse were calculated using the Anisotropic Analytical Algorithm (AAA), while the automated plans in MOZI were calculated using the Monte Carlo (MC) algorithm with a statistical uncertainty of 1% per plan. The same beam geometry, arc arrangement, collimator settings, and control‐point configuration were used for all planning approaches. Specifically, all plans used four full VMAT arcs with identical gantry angle ranges, collimator angles, and 180 control points per arc.

### 2.2 Dose prediction

The dose prediction method employed in this study was a “dose‐band prediction” model previously proposed by Liu et al. ^21^. This model utilizes parallel upper‐ and lower‐bound convolutional neural networks (CNNs) to predict a range of achievable radiotherapy doses, thereby enhancing the flexibility and robustness of automated planning and plan quality assurance. The 70 treatment plans were randomly divided into three groups: 50 for model training (training set), 10 for dose prediction validation (validation set), and 10 for testing the performance of both the dose prediction model and the automated planning workflow (test set).

### 2.3 Framework of auto‐planning

The VP framework was implemented in the MOZI TPS through its Python scripting interface. The framework consisted of deterministic rule‐based components and an LLM‐based advisory module. Auxiliary structure generation, template initialization, dose‐metric extraction, safety validation, and plan execution were performed using predefined Python scripts. The LLM was used to interpret structured dosimetric feedback and propose adjustments to existing optimization parameters; it did not call TPS tools or directly execute plan modifications.

During preprocessing, the VP automatically generated auxiliary structures using predefined geometric rules, including 5‐mm and 10‐mm PTV expansion rings, OAR structures trimmed from the PTV by 2 mm, and a small intestine–PTV intersection structure. The planning template contained fixed constraint functions and initial weights, whereas the optimization values differed between initialization strategies. For protocol‐based initialization, OAR objectives were derived from RTOG and institutional population‐level dose limits. For prediction‐guided initialization, the dose‐band prediction model generated a patient‐specific three‐dimensional dose grid from the planning CT and PTV/OAR contours. Following prior work,^21^the lower dose band (*λ* = 80) was used for prediction‐guided OAR objective initialization. A Python script calculated the relevant dose‐volume metrics directly from the predicted dose grid and assigned these exact patient‐specific values to the corresponding existing objectives. PTV objectives remained anchored to the prescription dose, and the 5‐mm and 10‐mm ring objectives were fixed at 90% and 80% of the prescription dose, respectively, to preserve target coverage and dose fall‐off. Initial objective weights were set to 100 for PTV and Body objectives and 30 for OAR objectives. This dose‐to‐objective conversion requires only scripting access to the dose grid and objective list and is therefore not specific to the MOZI TPS. The complete initialization template and a patient‐level comparison between protocol‐based and prediction‐guided parameters are provided in Supplementary Appendix B.

After initialization, the VP performed plan optimization and dose calculation using the Monte Carlo (MC) algorithm in the MOZI TPS. The resulting plan state was converted into structured numerical feedback for the LLM. At each iteration, the LLM received the clinical goal, achieved dose, current objective value and weight, penalty score, penalty proportion, historical adjustment information, and goal‐achievement status for each ROI.

The LLM used in this study was GLM‐4.1V‐9B‐Thinking, ^22^ accessed through its application programming interface. The system prompt assigned the model the role of a senior radiotherapy physicist and specified the planning objective, adjustment rules, permitted actions, and required JSON output format. The output schema included the ROI, constraint function, current and adjusted volumes, current and adjusted optimization values, and current and adjusted weights. The volume parameter was used for objective identification and was required to remain unchanged. The LLM was permitted only to modify the optimization values and weights of existing objectives; it could not create new objectives, constraints, or structures. Representative system and iterative user prompt templates are provided in Supplementary Appendix A.

Optimization proceeded in two stages. In the constraint‐satisfaction stage, the LLM prioritized achieving the required PTV coverage and OAR constraints by reducing objective values or increasing objective weights. In the refinement stage, after all predefined criteria had been satisfied, the LLM attempted to further reduce OAR doses. After each LLM recommendation, the VP parsed the structured output and applied the validated parameter changes through the Python scripting interface, followed by re‐optimization and dose calculation. This formed a fully automated perception–decision–execution loop.

Before execution, every LLM‐generated recommendation was screened by a deterministic rule‐based safety engine. A recommendation was rejected if it (i) inappropriately increased an upper‐bound OAR objective, (ii) assigned a weight outside the MOZI‐supported range of 1–1000, (iii) set an OAR optimization value above its predefined clinical safety limit, or (iv) contained invalid JSON, missing fields, invalid data types, or a reference to a nonexistent ROI or constraint. Rejected recommendations were not executed, and the plan state remained unchanged. The safety engine generated structured corrective feedback describing the violation and appended this feedback to the context of the next LLM interaction.

The workflow executed up to 20 optimization cycles and terminated early only when the LLM returned [stop] and the program independently verified that all predefined PTV and OAR criteria had been satisfied. All non‐LLM components of the workflow, including data extraction, safety validation, and TPS execution, were deterministic.

Figure 1 illustrates the workflow of the proposed intelligent automatic radiotherapy planning framework VP.

**FIGURE 1:**
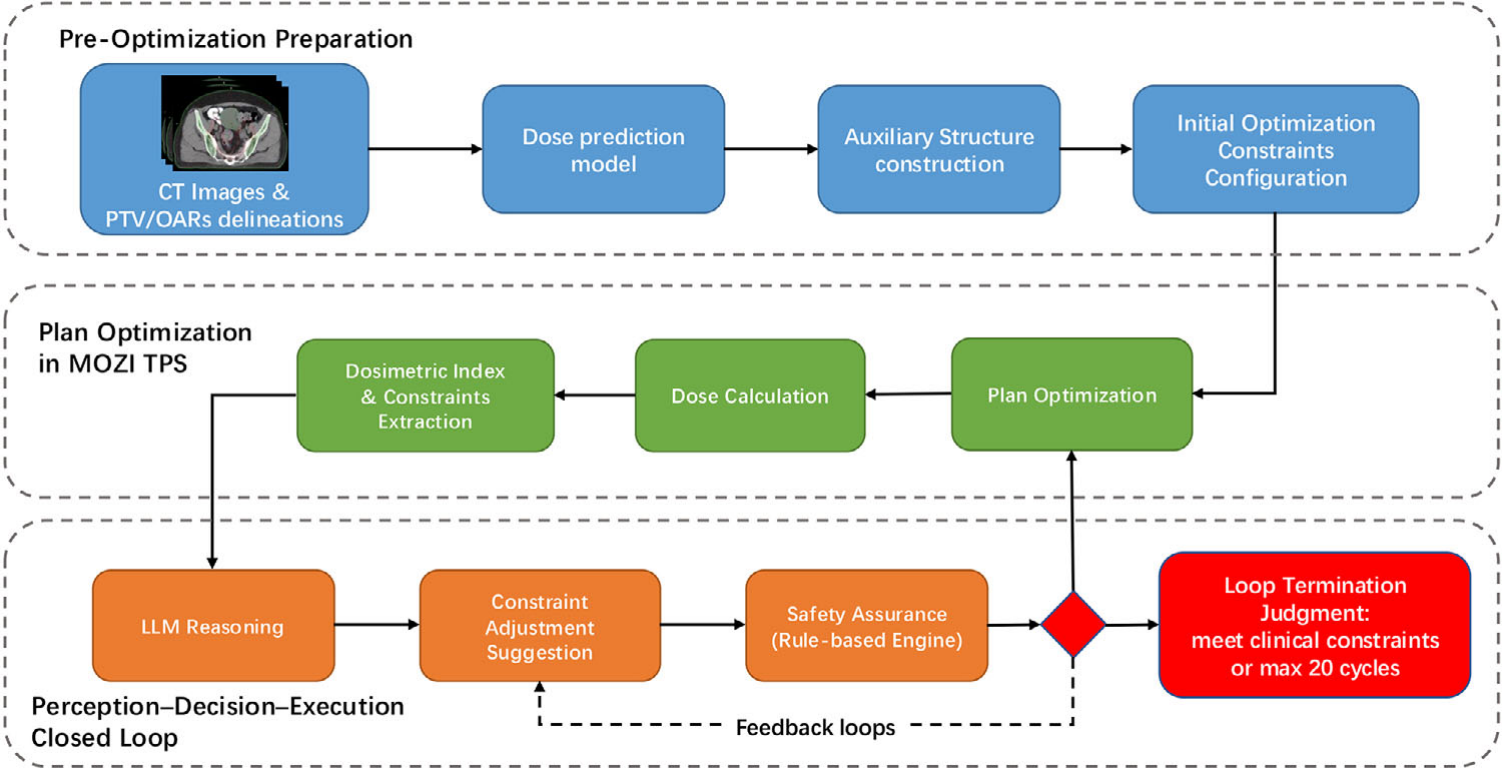
Workflow of the proposed VP—driven automatic radiotherapy planning framework.

To evaluate whether the proposed dose prediction– and large language model–based approach could generate flexible and high‐quality automatic treatment plans, the automatically generated plans were compared with the corresponding manually created clinical plans originally generated in the Eclipse TPS and used for patient treatment, as well as with automated plans produced under the following three scenarios:

- Dose prediction only (DP‐only method)
  For 10 test patients, the dose prediction model was used to estimate achievable doses for the PTV and OARs. The TPS automatically applied these predicted doses to preset optimization constraints and initiated plan optimization; however, no LLM was involved in parameter adjustment during the optimization process.
- Template and LLM (LLM‐only method)
  Optimization parameters were preset in the TPS according to the physician's prescription, using the same predefined template for all 10 patients. During plan optimization, the TPS dynamically adjusted the parameters by invoking the LLM via the VP framework.
- Use both dose prediction and LLM (DP + LLM method)
  This scenario represented the full VP workflow described above. The TPS used the dose prediction results to perform patient‐specific initialization of optimization constraints based on each patient's CT images and anatomical structures. During optimization, the LLM was accessed through the VP interface to dynamically refine the optimization constraints.

### 2.4 Performance evaluation

- Evaluations of dose prediction
  The accuracy of the dose prediction model was assessed by comparing the predicted upper‐band, middle‐line, and lower‐band doses with the reference clinical doses manually generated in the Eclipse TPS. The absolute percentage dose difference, $\delta |D_{i}|$, was calculated as:
(1) $$\delta \left|D_{i}\right|=\frac{\left|D_{i\;predicted}-D_{i\;reference}\right|}{Prescription\;Dose}*100\%$$
  Model evaluation was performed using a 10‐patient test set independent of the training and validation cohorts. Additionally, dose–volume histograms (DVHs) of the reference dose and predicted dose bands were presented for one representative patient to illustrate the model performance.
- Evaluation metrics of auto‐planning
  To assess the performance of automated planning, dosimetric and plan complexity indices of automatic plans generated under the three strategies were compared with the corresponding manually generated clinical plans for patients in the test set.

#### 2.4.1 Dosimetric metrics

For the PTVs, all treatment plans were normalized to identical target coverage before comparison, such that the PTV D95% was equal to 100% of the prescribed dose (i.e., 95% of the PTV received at least the prescribed dose). The dose–volume histogram (DVH) was used to assess the dose distributions of the PTV and OARs. The PTV evaluation metrics included the mean PTV dose (Dmean), homogeneity index (HI), conformity index (CI), and gradient index (GI), which were calculated as follows^23^, ^24^

(2) $$\mathrm{HI}=\frac{D_{2\%}-D_{98\%}}{D_{50\%}}$$

(3) $$\mathrm{CI}=\frac{{\left(V_{PTV}\cap V_{pres}\right)}^{2}}{V_{PTV}\times V_{pres}}$$

(4) $$\mathrm{GI}=\frac{V_{\frac{1}{2}pres}}{V_{pres}}$$

where D_2%_, D_98%_ and D_50%_ are the doses values covering 2%, 98%, and 50% of the PTV volume from DVH respectively; V_PTV_ is the volume of PTV; V_pres_ is the volume covered by the prescribed dose; V_PTV_∩V_pres_ is the volume of the PTV covered by the prescribed dose; V_1/2pres_ is the volume covered by 50% of the prescribed dose. A smaller HI indicates a more uniform dose distribution within the PTV, a CI closer to 1 reflects higher conformity of the plan, and a smaller GI corresponds to a steeper dose fall‐off outside the target.

For OARs, the evaluated metrics included the D_mean_ of bladder, rectum, and small intestine, the D_50%_ of bladder and rectum, the maximum radiation dose received by small intestine with a volume of 2 cc (D_2cc_), the D_5%_ of left and right Femoral Head, and the maximum radiation dose received by spinal cord with a volume of 0.1 cc (D_0.1cc_).

#### 2.4.2 Complexity metrics

Treatment plans were first exported from the TPS in Digital Imaging and Communications in Medicine (DICOM) format, and a Python script was subsequently used to calculate various plan complexity metrics from the plan files. Complexity parameters were computed for each control point and then aggregated across the plan using a weighted sum based on the monitor units (MUs) at each control point. For the Halcyon linear accelerator, which is equipped with a dual‐layer multi‐leaf collimator (MLC), both proximal and distal MLC banks operate cooperatively for beam modulation. Based on a previous study conducted at our center,^25^four plan complexity metrics were calculated for each MLC layer, as summarized in Table 1. Higher MU and CA values, and lower BA and MCS values, indicate greater plan complexity.

**TABLE 1:** Complexity metrics of dual‐layer MLCs and their corresponding references.

Complexity metrics	Reference
Monitor unit (MU)	26
Beam area of distal MLC (d‐BA)	26
Beam area of proximal MLC (p‐BA)	26
Circumference/area of distal MLC (d‐C/A)	27
Circumference/area of proximal MLC (p‐C/A)	27
Modulation complex score of distal MLC (d‐MCS)	28
Modulation complex score of proximal MLC (p‐MCS)	28

### 2.5 Statistical analysis

The mean value and standard deviation (SD) were presented as mean ± SD for each dosimetric metric and plan complexity metric value. Given the small size of the independent test cohort (*n* = 10) and the limited reliability of normality testing in small samples, statistical comparisons between the DP + LLM strategy and each of the other planning strategies were performed using two‐sided exact Wilcoxon signed‐rank tests. This nonparametric paired test does not require the paired differences to follow a normal distribution. *P* values of < 0.05 were considered statistically significant. All analyses, including the extraction of plan complexity metrics, calculation of means, SDs, and *P* values, were performed using Python 3.10.9.

## 3 RESULTS

### 3.1 Performance of dose prediction

The mean absolute percentage dose differences, $\delta |D_{i}|$, are summarized in Table 2. Dose–volume histograms (DVHs) of the reference dose and the predicted dose bands for a representative patient are presented in Figure 2.

**TABLE 2:** The absolute percentage dose differences between reference dose and Upper‐band dose, Middle‐line dose, and Lower‐band dose with *λ* = 80.

Structures	Indices	Upper‐band dose (%)	Middle‐line dose (%)	Lower‐band dose (%)
PTV	D_mean_	1.61 ± 0.58	0.64 ± 0.41	2.46 ± 0.79
	D_95%_	3.28 ± 0.51	2.11 ± 0.5	0.84 ± 0.43
	D_98%_	7.35 ± 1.27	5.2 ± 1.19	2.79 ± 0.89
Bladder	D_mean_	0.71 ± 0.25	0.54 ± 0.23	1.8 ± 0.23
	D_50%_	0.9 ± 0.53	0.83 ± 0.45	2.41 ± 0.6
Rectum	D_mean_	1.61 ± 0.58	0.64 ± 0.41	2.46 ± 0.79
	D_50%_	2.15 ± 1.41	1.19 ± 0.94	2.37 ± 1.37
Small Intestine	D_mean_	0.71 ± 0.24	0.46 ± 0.25	1.64 ± 0.28
	D_2cc_	1.92 ± 0.55	1.29 ± 0.57	0.7 ± 0.52
Femoral Head L	D_5%_	0.78 ± 0.49	1.78 ± 0.95	3.29 ± 0.99
Femoral Head R	D_5%_	0.98 ± 0.86	1.37 ± 1.27	2.66 ± 1.54
Spinal cord	D_0.1cc_	4.08 ± 2.48	6.78 ± 2.76	9.52 ± 2.99

**FIGURE 2:**
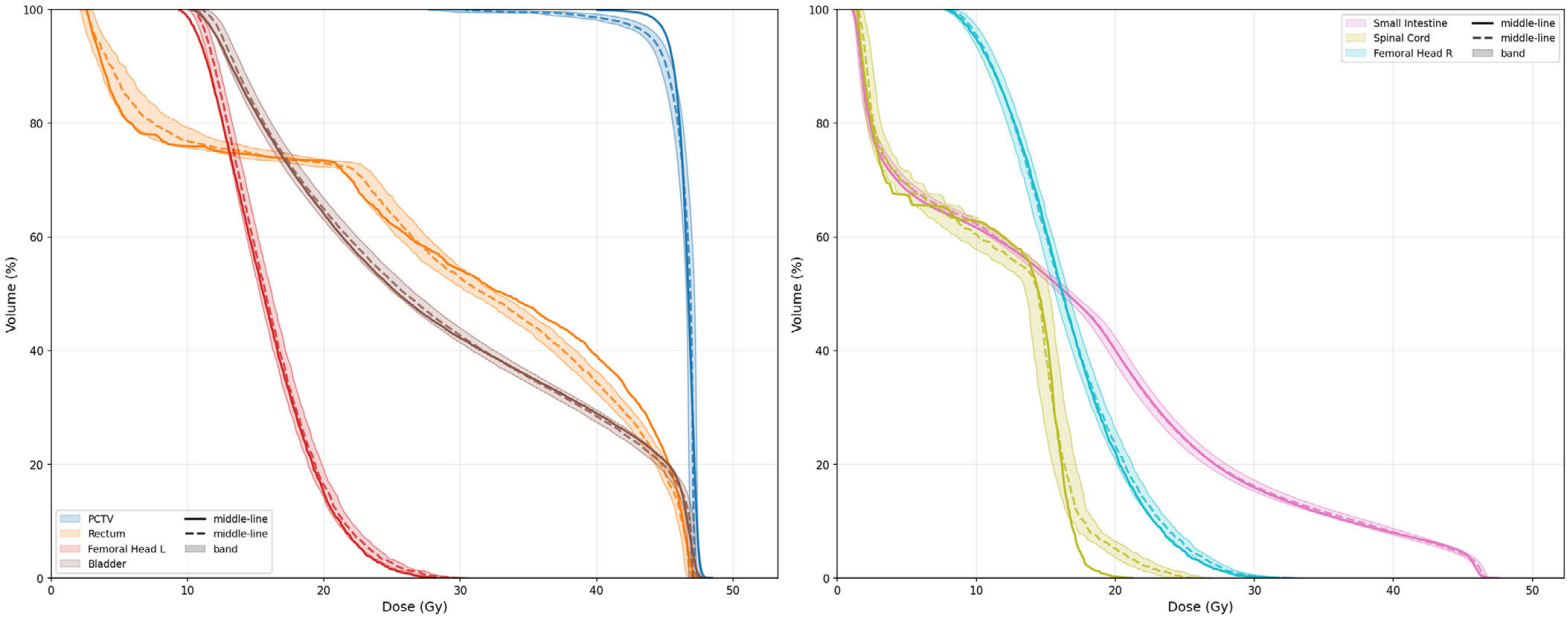
DVHs of reference dose and predicted dose band for one representative case.

### 3.2 Dosimetric results

Table 3 summarizes the dosimetric metrics and corresponding *P* values for the PTV and OARs in 10 cervical cancer patients. For clearer visualization, Figure 3 presents the dose values and associated errors for OARs, while Figure 4 illustrates the isodose distributions, dose difference maps, and dose–volume histograms (DVHs) for a representative patient, comparing the four types of treatment plans. To minimize bias from differing prescription doses, all dosimetric values are reported as percentages of the prescription dose.

**TABLE 3:** Dosimetric results of automatic plans generated using the DP + LLM, DP‐only, and LLM‐only methods, along with the corresponding manually generated plans.

Structures	Indices	DP + LLM	DP‐only (*P* value)	LLM‐only (*P* value)	Manua (*P* value)
PTV	D_mean_ (%)	**103.46 ± 0.73**	103.53 ± 2.35 (0.6250)	103.43 ± 0.80 (1.0000)	103.34 ± 0.22 (0.6953)
	HI	**0.07 ± 0.01**	0.06 ± 0.02 (0.4316)	0.07 ± 0.01 (0.5566)	0.06 ± 0.00 (0.0645)
	CI	**0.92 ± 0.01**	0.88 ± 0.06 (**0.0488**)	0.94 ± 0.01 **(9.7656 × 10^−3^)**	0.93 ± 0.01 (0.3223)
	GI	**3.65 ± 0.13**	3.73 ± 0.33 (0.4922)	3.72 ± 0.17 (0.1602)	3.66 ± 0.12 (1.0000)
Bladder	D_mean_ (%)	**65.00 ± 4.29**	70.06 ± 4.55 (**1.9531× 10^−3^**)	68.05 ± 4.22 **(0.0195)**	73.37 ± 4.46 (**1.9531× 10^−3^**)
	D_50%_ (%)	**64.82 ± 9.05**	73.04 ± 8.97 (**1.9531× 10^−3^**)	69.44 ± 8.97 **(9.7656 × 10^−3^)**	74.32 ± 8.00 (**1.9531× 10^−3^**)
Rectum	D_mean_ (%)	**70.12 ± 4.39**	72.67 ± 5.86 **(0.0273)**	70.87 ± 2.04 (0.9219)	74.60 ± 5.93 **(9.7656 × 10^−3^)**
	D_50%_ (%)	**74.94 ± 8.25**	80.88 ± 8.01 (**9.7656 × 10^−3^**)	78.87 ± 4.43 (0.0840)	81.69 ± 7.95 (**0.0137**)
Small Intestine	D_mean_ (%)	**40.20 ± 3.42**	40.66 ± 3.88 (0.2324)	42.04 ± 3.15 (**0.0273**)	40.76 ± 3.09 (0.2324)
	D_2cc_ (%)	**104.07 ± 0.36**	105.60 ± 3.70 (1.0000)	103.99 ± 0.31 (0.4316)	103.70 ± 0.67 (0.0840)
Femoral Head L	D_5%_ (%)	**48.17 ± 7.45**	74.72 ± 5.28 (**1.9531 × 10^−3^**)	52.61 ± 8.69 (**0.0273**)	57.45 ± 9.98 (**0.0488**)
Femoral Head R	D_5%_ (%)	**46.41 ± 7.48**	74.01 ± 4.62 (**1.9531 × 10^−3^**)	55.96 ± 7.41 (**5.8594 × 10^−3^**)	59.04 ± 11.40 (**0.0371**)
Spinal cord	D_0.1cc_ (%)	**45.21 ± 11.24**	55.97 ± 3.73 (**0.0137**)	65.16 ± 4.59 (**3.9062 × 10^−3^**)	40.49 ± 5.99 (0.3223)

*Note*: *P* values from two‐sided exact Wilcoxon signed‐rank tests comparing each of the other three planning strategies with the DP + LLM plans are also shown. All dosimetric values are reported as percentages of the prescription dose.

**FIGURE 3:**
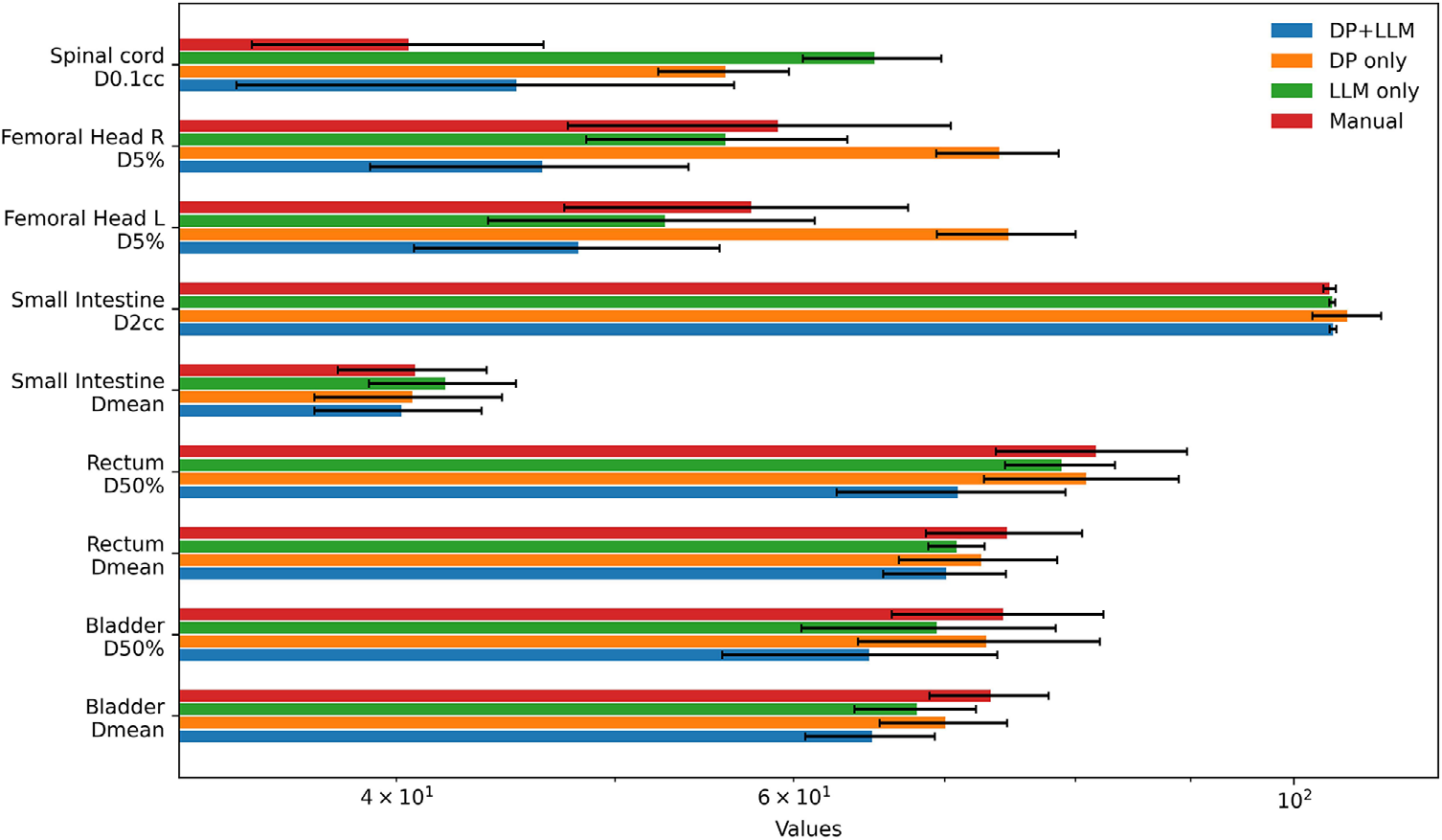
Dosimetric metrics of OARs in four different planning strategies.

**FIGURE 4:**
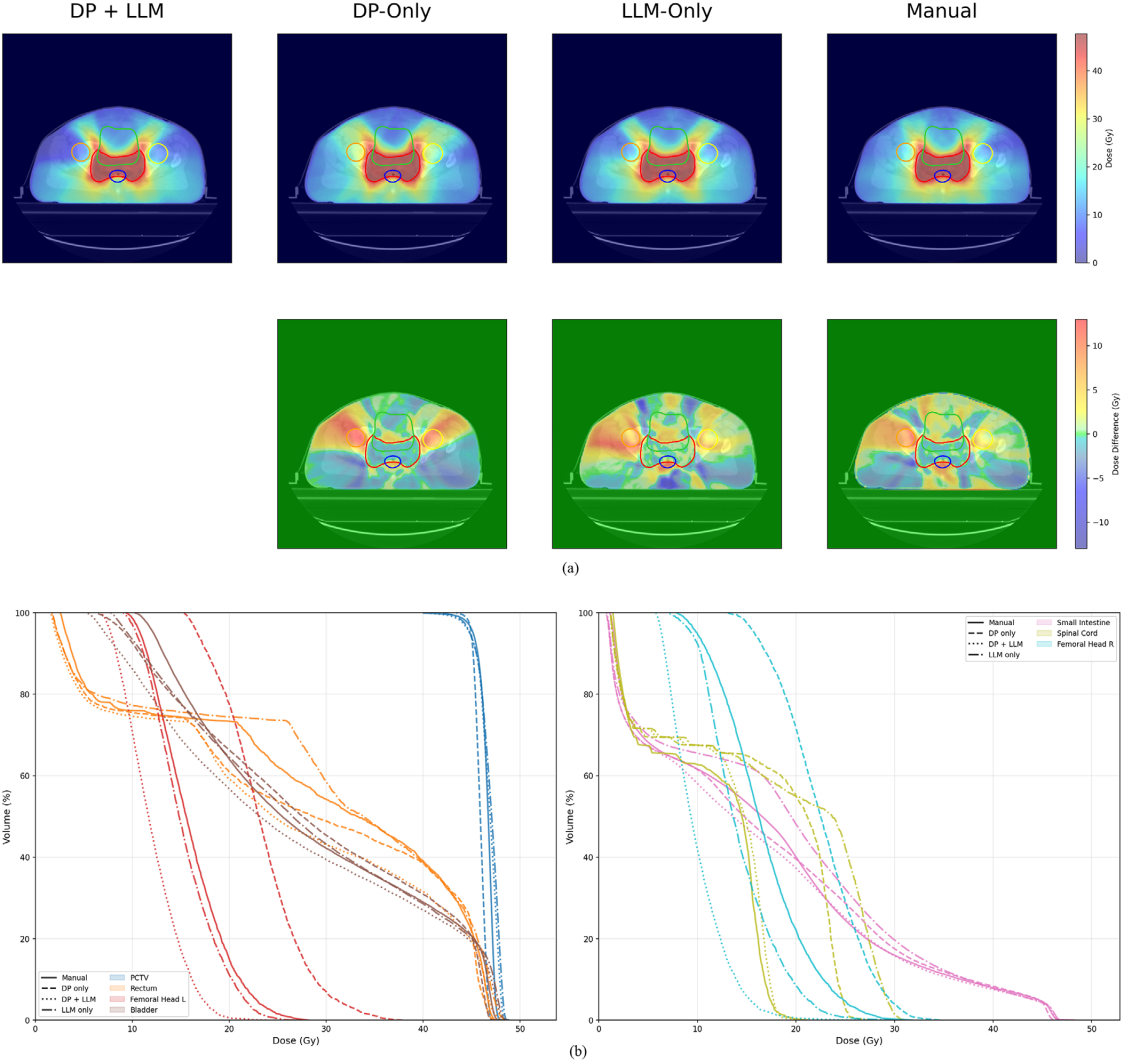
(a) Isodose distribution and (b) DVH of four different kinds of treatment plans for one representative patient.

No significant differences in PTV Dmean, HI, or GI were observed between the DP + LLM plans and any of the other three planning strategies. Compared with DP + LLM, CI was significantly lower for DP‐only (*P* = 0.0488) and significantly higher for LLM‐only (*P* = 9.7656 × 10^−^^3^), whereas no significant difference was observed for manual plans (*P* = 0.3223). For the bladder (Dmean and D50%) and bilateral femoral heads (D5%), the DP + LLM plans achieved significantly lower doses than all three other planning strategies. For the rectum, the DP + LLM plans yielded the lowest Dmean and D50% among the four approaches, with significant differences relative to the DP‐only and manual plans but not the LLM‐only plans. The small intestine Dmean was also lowest for the DP + LLM plans, although the difference reached statistical significance only versus the LLM‐only plans. The small intestine D2cc obtained with the DP + LLM method was higher than that of the manual and LLM‐only plans, but these differences were not statistically significant. Similarly, the DP + LLM plans resulted in a higher spinal cord D0.1cc than the manual plans without statistical significance, while this value was significantly lower than those of the DP‐only and LLM‐only plans (*P* < 0.05).

### 3.3 Complexity metrics

Table 4 and Figure 5 present the comparisons of plan complexity metrics and the corresponding *P* values for the 10 treatment plans. For ease of interpretation, each complexity metric in Table 4 is annotated with arrows: ↑ denotes a positive correlation between the metric value and plan complexity (i.e., larger values indicate higher complexity), whereas ↓ denotes an inverse correlation. Overall, the DP + LLM strategy generated automatic plans with complexity comparable to those of manual plans, while exhibiting higher complexity than the other two automatic planning strategies, reflecting enhanced modulation capability for improved OAR sparing. The DP + LLM plans yielded the highest monitor unit (MU) values among the four approaches; however, no statistically significant difference was observed when compared with the manual plans. For the BA‐, C/A‐, and MCS‐related parameters, layer‐specific analyses of MLC revealed that manual plans exhibited significantly greater complexity in the distal MLC than the DP + LLM plans, whereas significantly lower complexity was observed in the proximal MLC. This phenomenon can be attributed to the fact that the DP + LLM strategy demonstrated relatively balanced complexity between the distal and proximal MLC layers, whereas the manual plans showed pronounced variability between the two layers.

**TABLE 4:** Complexity metrics of the automatic plans generated by the DP+LLM, DP‐only and LLM‐only methods, along with the corresponding manually generated plan.

Metrics	DP + LLM	DP‐only (*P* value)	LLM‐only (*P* value)	Manual (*P* value)
MU↑	**808.00 ± 41.34**	786.37 ± 31.90 (0.0840)	767.79 ± 22.17 (**0.0371**)	771.01 ± 78.56 (0.2324)
d‐BA↓	**97.02 ± 6.00**	103.30 ± 5.63 (**1.9531 × 10^−3^**)	101.77 ± 7.01 (**0.0137**)	94.99 ± 16.33 (0.4316)
p‐BA↓	**96.25 ± 6.06**	102.94 ± 5.88 (**1.9531 × 10^−3^**)	101.03 ± 7.43 **(9.7656 × 10^−3^)**	123.61 ± 15.12 (**1.9531 × 10^−3^**)
d‐C/A↑	**1.20 ± 0.06**	1.17 ± 0.05 (0.1309)	1.14 ± 0.05 (0.1055)	1.44 ± 0.17 (**5.8594 × 10^−3^**)
p‐C/A↑	**1.23 ± 0.06**	1.19 ± 0.06 (**0.0273**)	1.17 ± 0.06 (0.0645)	1.09 ± 0.08 (**1.9531 × 10^−3^**)
d‐MCS↓	**0.42 ± 0.04**	0.42 ± 0.03 (0.6250)	0.44 ± 0.02 (0.1309)	0.35 ± 0.04 (**1.9531 × 10^−3^**)
p‐MCS↓	**0.41 ± 0.03**	0.42 ± 0.03 (0.1309)	0.43 ± 0.02 (0.0840)	0.43 ± 0.04 (**0.0273**)

*Note*: *P* values from two‐sided exact Wilcoxon signed‐rank tests comparing each of the other three planning strategies against the DP + LLM plans are also reported.

**FIGURE 5:**
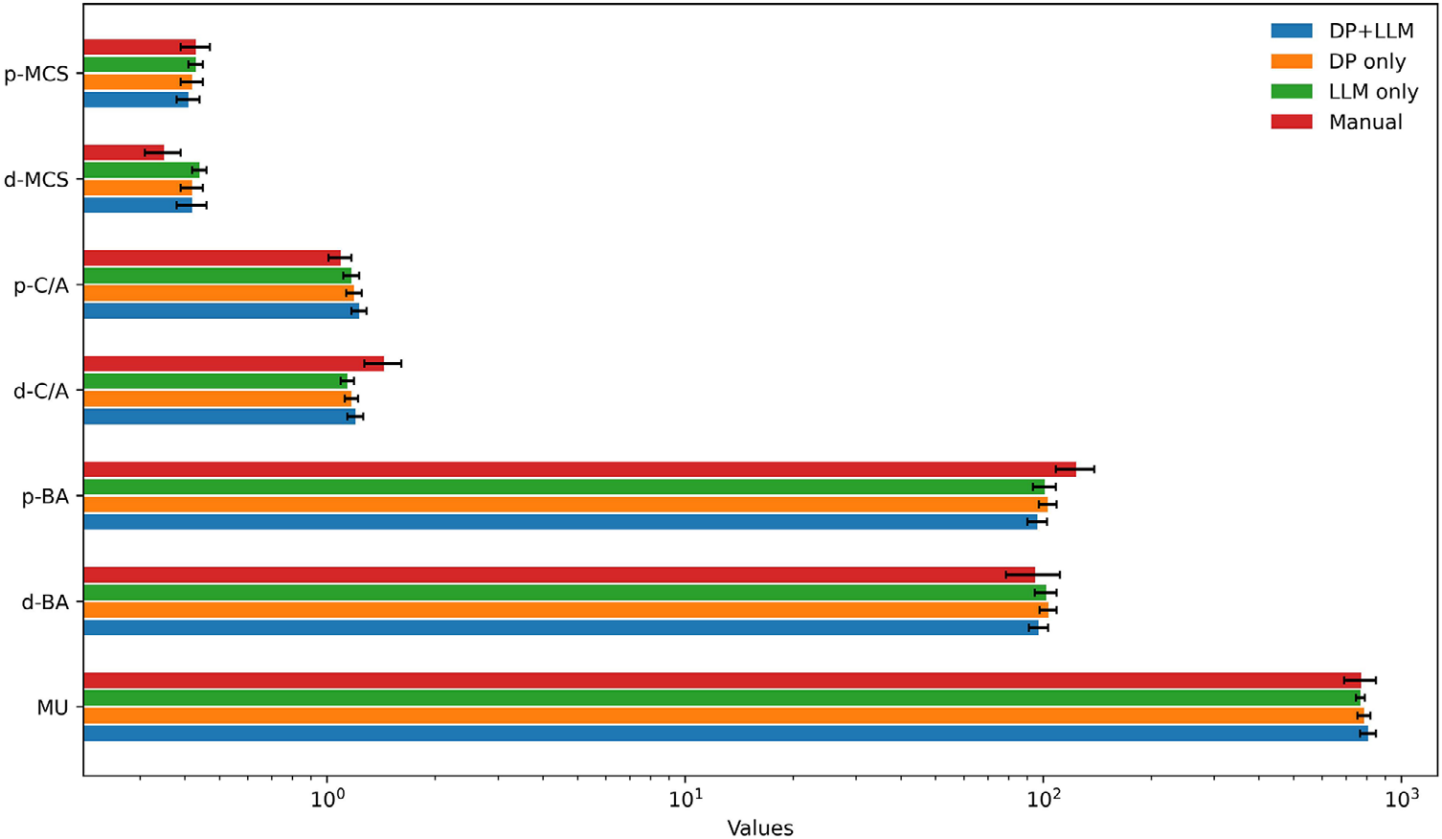
Complexity metrics of treatment plans for the four different planning strategies.

In addition, manual plans exhibited greater variability, as reflected by larger error bars, across all evaluated complexity metrics compared with the three automated planning strategies. This result suggests that, relative to human‐generated plans, automated approaches provide improved consistency in plan complexity.

## 4 DISCUSSION

In this study, we proposed and systematically validated a fully automated treatment planning framework that integrates a dose prediction model with an LLM, termed the VP. By incorporating patient‐specific dose priors with an intelligent optimization strategy guided by real‐time dosimetric feedback, the proposed framework achieved improved sparing of OARs and enhanced plan consistency, while maintaining adequate target coverage and plan deliverability.

Recently, Maniscalco et al. established the feasibility of end‐to‐end autonomous radiotherapy planning using a multimodal compound AI platform that integrated seven specialized LLM agents, directive‐conditioned three‐dimensional dose prediction, retrieval‐augmented institutional knowledge, and commercial TPS operations across multiple disease sites and treatment techniques.^20^Although both studies integrate dose prediction with LLM‐driven optimization, their scopes and primary objectives differ. The study by Maniscalco et al. focused on developing a site‐agnostic, multi‐agent autonomous planning platform and evaluating it against clinical reference plans. In contrast, the present study performed a controlled component‐level comparison of DP‐only, LLM‐only, and combined DP + LLM strategies within the same TPS and patient cohort. This design allowed us to isolate the individual and synergistic effects of dose prediction and LLM‐based adaptive optimization. Furthermore, the present study incorporated a rule‐based safety engine to intercept clinically inappropriate LLM recommendations and evaluated dual‐layer MLC complexity, providing additional insight into plan deliverability and modulation behavior.

Comparative analyses indicated that relying solely on dose prediction (DP‐only) or exclusively on a large language model (LLM‐only) was insufficient to consistently achieve optimal plan quality across all evaluation dimensions. The DP‐only approach effectively leveraged patient‐specific dose priors to provide reasonable initialization of optimization constraints; however, its lack of adaptive parameter adjustment during the optimization process limited its ability to handle complex multi‐objective trade‐offs. In contrast, the LLM‐only approach offered greater flexibility by dynamically tuning optimization parameters based on dosimetric feedback and generally outperformed the DP‐only strategy in terms of dosimetric outcomes. Nevertheless, its dependence on a unified optimization template during initialization restricted its capacity to fully accommodate inter‐patient anatomical variability, thereby reducing optimization efficiency and overall robustness.

By contrast, the DP + LLM strategy effectively integrated the complementary strengths of both approaches. The dose‐band prediction model provided conservative, patient‐specific OAR dose constraints during the initial optimization stage, steering the solution toward a clinically favorable region of the search space. Subsequently, the LLM dynamically adjusted optimization objectives and weights based on real‐time dosimetric feedback from successive optimization cycles, enabling fine‐grained balancing between target coverage and OAR sparing. This synergistic mechanism was directly reflected in the dosimetric outcomes. Compared with the other two automated planning strategies, the DP + LLM plans achieved significantly improved dose sparing for several critical OARs, including the bladder, rectum, and bilateral femoral heads, and outperformed the clinically delivered manual plans for most OAR‐related metrics. Importantly, these improvements were achieved while maintaining PTV coverage, homogeneity, conformity, and dose gradient metrics comparable to those of the manual plans, indicating that enhanced OAR protection was not obtained at the expense of target dose quality. However, the comparison between the automated plans and the manually generated clinical plans should be interpreted with caution because these plans were generated using different TPS platforms and dose calculation algorithms. Specifically, the manual plans were generated in Eclipse using AAA, whereas all automated plans were generated in MOZI using an MC algorithm. Thus, the observed dosimetric differences may partly reflect differences in dose calculation and the interaction between each dose engine and its corresponding optimization system, rather than the planning strategy alone. Although the manual plans provide a clinically meaningful reference because they were the approved plans used for patient treatment, they do not constitute an algorithm‐matched control.

In terms of plan complexity, this study further elucidated differences in dose modulation patterns among the planning strategies through a detailed analysis of dual‐layer MLC complexity metrics. Overall, treatment plans generated using the DP + LLM strategy exhibited complexity levels comparable to those of the manual plans, while being more complex than those produced by the DP‐only and LLM‐only strategies. This observation suggests that, relative to the other two automatic approaches, the DP + LLM strategy introduced finer and more adaptive dose modulation to achieve improved OAR sparing. Notably, in manual plans, layer‐specific analyses revealed distinct modulation behaviors between the proximal and distal MLC banks. Manual plans demonstrated significantly higher complexity in the distal MLC layer, accompanied by significantly lower complexity in the proximal layer, indicating an imbalanced modulation pattern across layers. In contrast, the DP + LLM strategy yielded a more balanced distribution of modulation complexity between the proximal and distal MLC layers, reflecting a more uniform utilization of the dual‐layer MLC system during optimization. However, it should be emphasized that, to date, no established guidelines or consensus frameworks exist for the joint evaluation of proximal and distal MLC complexity in dual‐layer MLC systems. Consequently, the interpretation of layer‐specific complexity remains an open challenge, and the development of standardized metrics and evaluation criteria for such systems represents an important direction for future research.

Furthermore, compared with the manually generated plans, all three automatic planning strategies exhibited reduced dispersion in plan complexity metrics, as evidenced by lower error‐to‐mean ratios. This observation indicates that the VP framework effectively decreased reliance on individual physicist and dosimetrist experience and subjective decision‐making, thereby mitigating inter‐case variability associated with human factors. Such enhanced consistency is particularly advantageous for large‐scale clinical deployment and multi‐center quality assurance, as it facilitates treatment plan standardization across patients and planners.

Despite the strong reasoning capabilities of LLMs, their application in treatment plan optimization remains susceptible to clinically unreasonable or logically inconsistent recommendations, primarily due to hallucinations and imperfect grounding in physical constraints.^19^, ^29^, ^30^To address this limitation, the VP framework incorporated a rule‐based engine that enforced multi‐level safety constraints on all LLM‐generated outputs prior to execution. This mechanism effectively intercepted operations that violate fundamental physical or clinical principles. By feeding corrective feedback back into subsequent LLM interactions, the framework further enhanced the clinical robustness and reliability of model outputs, providing a feasible pathway for the safe integration of LLMs into automated radiotherapy planning workflows. A deterministic rule‐based safety engine was selected instead of a second LLM‐based safety agent to reduce cascading model uncertainty and improve reproducibility.

Despite its strengths, several limitations of this study should be acknowledged. First, the validation cohort was derived from a single institution, a single disease site (cervical cancer), and a specific linear accelerator platform, which may limit the generalizability of the results to other tumor sites, treatment techniques, and delivery systems. In addition, the independent test cohort included only 10 patients, which limited the statistical power and precluded reliable verification of the distributional assumptions required for parametric testing. Although exact Wilcoxon signed‐rank tests were used to reduce the reliance on normality assumptions, the statistical findings should be interpreted as exploratory and require validation in larger independent cohorts. Future studies should consider alternative data allocations, external validation cohorts, or multi‐institutional datasets to improve statistical precision and generalizability. Second, the safety rules implemented in the rule‐based engine were tailored to the current study scenario and may require adaptation or extension to accommodate more diverse clinical cases and planning objectives. Moreover, although all non‐LLM components of the VP workflow were deterministic, the LLM itself may generate different adjustment trajectories across repeated runs. Repeated‐run reproducibility was not evaluated in this feasibility study and should be assessed in future work before clinical deployment. Third, the manual and automated plans were generated using different TPS platforms and dose calculation algorithms (Eclipse AAA versus MOZI MC). Therefore, the comparison with manual plans may be confounded by platform‐ and dose‐engine–related differences. Furthermore, this work represents a feasibility study of the combined dose prediction and LLM‐driven planning strategy. Neither the MOZI treatment planning system nor the proposed VP framework has yet been deployed in routine clinical practice. Future investigations should include multi‐institutional validation, patient‐specific quality assurance, and clinical toxicity outcomes to more comprehensively assess the clinical robustness and translational value of the proposed approach.

## 5 CONCLUSION

In conclusion, this study demonstrates that integrating dose prediction models with LLM‐driven dynamic optimization provides an efficient, robust, and clinically promising solution for fully automated radiotherapy treatment planning. By combining patient‐specific prior information with feedback‐aware decision‐making during iterative optimization, the proposed hybrid intelligent framework advances planning automation in terms of plan quality, consistency, and safety. These findings suggest a feasible pathway toward intelligent, standardized, and scalable radiotherapy planning workflow.

## AUTHOR CONTRIBUTIONS

Heling Zhu wrote the manuscript and prepared the tables and figures. Junqiang Li and Wei Zhang analyzed the data and built the dose‐band prediction model. Heling Zhu and Yongguang Liang contributed to the acquisition of data. Heling Zhu, Yiming Zhang and Wenbo Li involved in the conception and design of the study. Bo Yang and Qichao Zhou supervised the study and provided administrative and funding support. All authors reviewed the manuscript.

## CONFLICT OF INTEREST STATEMENT

The authors declare no conflicts of interest.

## ETHICS STATEMENT

This study was conducted in accordance with the Declaration of Helsinki (as revised in 2013) and was approved by the institutional ethics board of the Peking Union Medical College Hospital (no. I‐24PJ1111).

## CONSENT STATEMENT

Consent to Participate declaration was obtained from all the patients.

## Supporting information



Supporting Information

Supporting Information

## Data Availability

Authors will share data upon request to the corresponding author.
