# Supplementary material for: A virtual physicist framework integrating dose prediction and large language models for automated treatment planning
Source: J Appl Clin Med Phys. 2026 Nov 5;27(11):e70904. doi: 10.1002/acm2.70904 (PMC13735221; doi:10.1002/acm2.70904)
Supplement: Supplementary file 2 [file ACM2-27-e70904-s002.docx]

**Supplementary Appendix B: Initialization Templates and Examples**

Supplementary Table S1. Protocol-based initialization template for a 4500-cGy prescription

| **ROI** | **Function** | **Volume** | **Dose (cGy)** | **Priority** |
| --- | --- | --- | --- | --- |
| Bladder | Max DVH | 45.00% | 4000.00 | 30 |
| Bladder | Upper gEUD | — | 3300.00 | 30 |
| Bladder_TRIM_PCTV_2mm | Upper gEUD | — | 2000.00 | 30 |
| Body1 | Max Dose | — | 4950.00 | 100 |
| Femoral Head L | Max DVH | 5.00% | 3500.00 | 30 |
| Femoral Head L | Upper gEUD | — | 2300.00 | 30 |
| Femoral Head R | Max DVH | 5.00% | 3500.00 | 30 |
| Femoral Head R | Upper gEUD | — | 2300.00 | 30 |
| PCTV | Min DVH | 95.00% | 4500.00 | 100 |
| PCTV_Ring_5mm | Max Dose | — | 4050.00 | 30 |
| PCTV_Ring_10mm | Max Dose | — | 3600.00 | 30 |
| Rectum | Max DVH | 45.00% | 4000.00 | 30 |
| Rectum | Upper gEUD | — | 3300.00 | 30 |
| Rectum_TRIM_PCTV_2mm | Upper gEUD | — | 2000.00 | 30 |
| SI_INTER_PCTV | Max Dose | — | 4700.00 | 30 |
| Small Intestine | Max Dose | — | 4700.00 | 30 |
| Small Intestine | Upper gEUD | — | 2500.00 | 30 |
| Spinal Cord | Max Dose | — | 3500.00 | 30 |

Supplementary Table S2. Example comparison between protocol-based and prediction-guided initialization for one patient

| **ROI** | **Function** | **Protocol-based dose (cGy)** | **Prediction-guided dose (cGy)** | **Difference (cGy)** |
| --- | --- | --- | --- | --- |
| Bladder | Max DVH | 4000.00 | 3397.22 | −602.78 |
| Bladder | Upper gEUD | 3300.00 | 3195.15 | −104.85 |
| Bladder_TRIM_PCTV_2mm | Upper gEUD | 2000.00 | 2684.49 | +684.49 |
| Body1 | Max Dose | 4950.00 | 4670.82 | −279.18 |
| Femoral Head L | Max DVH | 3500.00 | 3513.42 | +13.42 |
| Femoral Head L | Upper gEUD | 2300.00 | 2645.25 | +345.25 |
| Femoral Head R | Max DVH | 3500.00 | 3463.13 | −36.87 |
| Femoral Head R | Upper gEUD | 2300.00 | 2659.43 | +359.43 |
| PCTV | Min DVH | 4500.00 | 4500.00 | 0 |
| PCTV_Ring_5mm | Max Dose | 4050.00 | 4050.00 | 0 |
| PCTV_Ring_10mm | Max Dose | 3600.00 | 3600.00 | 0 |
| Rectum | Max DVH | 4000.00 | 4064.46 | +64.46 |
| Rectum | Upper gEUD | 3300.00 | 3559.45 | +259.45 |
| Rectum_TRIM_PCTV_2mm | Upper gEUD | 2000.00 | 2898.16 | +898.16 |
| SI_INTER_PCTV | Max Dose | 4700.00 | 4670.82 | −29.18 |
| Small Intestine | Max Dose | 4700.00 | 4615.33 | −84.67 |
| Small Intestine | Upper gEUD | 2500.00 | 2014.56 | −485.44 |
| Spinal Cord | Max Dose | 3500.00 | 2631.57 | −868.43 |

The constraint functions and initial weights were identical between the two initialization strategies. The protocol-based template used population-level limits, whereas the prediction-guided template used patient-specific values calculated from the predicted three-dimensional dose grid. Prediction-guided values could be either more stringent or less stringent than protocol-based values depending on the patient’s anatomy. PTV and ring objectives remained prescription-anchored in both strategies to preserve target coverage and dose fall-off.
